# Experimental data on compressive strength and ultrasonic pulse velocity properties of sustainable mortar made with high content of GGBFS and CKD combinations

**DOI:** 10.1016/j.dib.2020.105961

**Published:** 2020-07-03

**Authors:** Hasan Sh Majdi, Ali Abdulhussein Shubbar, Mohammed Salah Nasr, Zainab S. Al-Khafaji, Hassnen Jafer, Muhammad Abdulredha, Zainab Al Masoodi, Monower Sadique, Khalid Hashim

**Affiliations:** a Department of Chemical Engineering and Petroleum Industries, Al‐Mustaqbal University College, Hillah, Iraq; b Department of Civil Engineering, Liverpool John Moores University, Henry Cotton Building, Webster Street, Liverpool, L3 2ET, UK; c Department of Civil Engineering, College of Engineering, University of Babylon, Babylon, Iraq; d Babylon Technical Institute, Al-Furat Al-Awsat Technical University, 51015, Babylon, Iraq; e Al-Furrat Al-Awsat Distribution Foundation, Ministry of Oil, Babylon, Iraq.; f Department of Civil Engineering, College of Engineering, University of Kerbala, Kerbala, Iraq; g Ministry of Construction and Housing, National Center for Construction Laboratories (NCCL), Babylon, Iraq.

**Keywords:** Cement replacement, high replacement level, compressive strength, ultrasonic pulse velocity

## Abstract

The development in the construction sector and population growth requires an increase in the consumption of construction materials, mainly concrete. Cement is the binder in concrete, so increasing cement production will increase the energy consumed, as well as in the emission of carbon dioxide. This harmful effect of the environment led to the search for alternative materials for cement, as the waste or by-products of other industries is a promising solution in this case. Among these common materials are ground granulated blast furnace slag (GGBS) and cement kiln dust (CKD). This dataset describes the compressive strength and ultrasonic pulse velocity of mortar consisted of high content of GGBS and CKD combinations as a partial substitute for cement (up to 80%) at the ages of 1, 2, 3, 7, 14, 21, 28, 56, 90 and 550 days. This dataset can help the researchers to understand the behaviour of GGBS and CKD in high replacement levels for cement during early (1 day) and later ages (550 days). According to this understanding, the authors believe that the data available here can be used to produce more environmentally friendly mortar or concrete mixtures by significantly reducing the amount of cement used by replacing it with waste or by-products of other industries.

## Specifications Table

**Table:** 

Subject	Civil engineering
Specific subject area	Building Materials, Concrete Technology, Mechanical and Durability Properties
Type of data	Tables, Figures and Images.
How data were acquired	Laboratory Experiments
Data format	Raw and Analysed
Parameters for data collection	Three different percentages of GGBS and CKD combinations are replaced the cement in a high levels (as well as the reference mixture without replacement) to produce sustainable mortar.
Description of data collection	Data was obtained from laboratory experiments at the ages of 1, 2, 3, 7, 14, 21, 28, 56, 90 and 550 days of compressive strength and ultrasonic pulse velocity properties of the hardened mortar
Data source location	Liverpool, United Kingdom
Data accessibility	The data are available within this article
Related research article	Shubbar, Ali Abdulhussein, Hassnen Jafer, Muhammad Abdulredha, Zainab S. Al-Khafaji, Mohammed Salah Nasr, Zainab Al Masoodi, and Monower Sadique. "Properties of cement mortar incorporated high volume fraction of GGBFS and CKD from 1 day to 550 days." Journal of Building Engineering (2020): 101327. https://doi.org/10.1016/j.jobe.2020.101327

## Value of the Data

• This data composed of alternative cement materials in the concrete industry for building construction.
• The information provided by this data are useful to find a significant solution to environmental problems through the re-use of industrial waste in new other applications as well as reducing the CO_2_ emissions that result from the cement industry.
• The data in this article is beneficial in producing sustainable mortar in which cement content is significantly reduced.
• This data helps others to understand the behaviour of hardened mortar containing high levels of GGBS and CKD during early and later ages.

## 1 Data Description

**Fig. 1:**
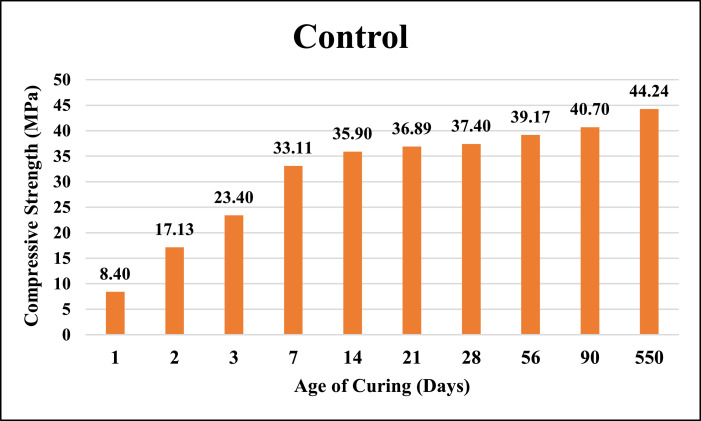
Average compressive strength of the Control mixture.

**Fig. 2:**
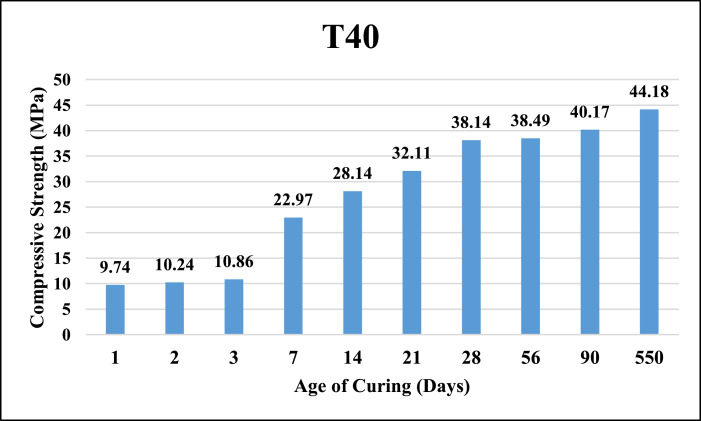
Average compressive strength of T40 mixture.

**Fig. 3:**
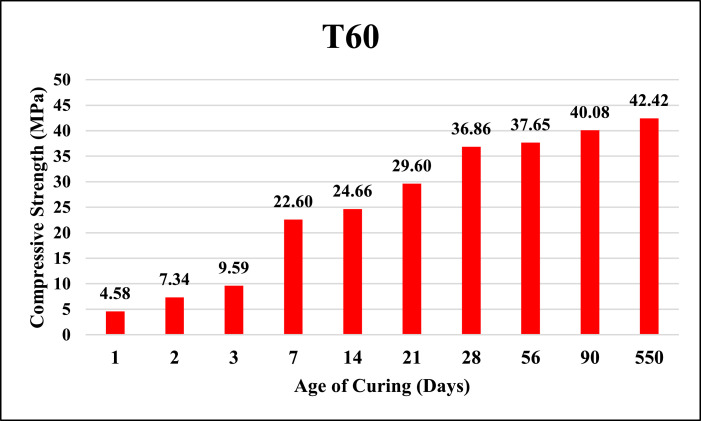
Average compressive strength of T60 mixture.

**Fig. 4:**
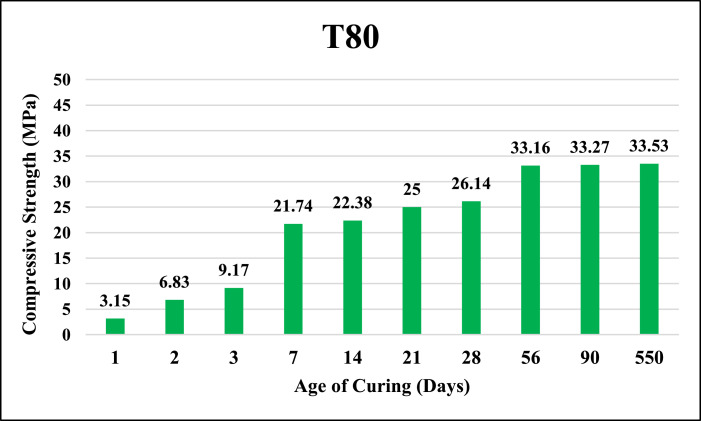
Average compressive strength of T80 mixture.

**Fig. 5:**
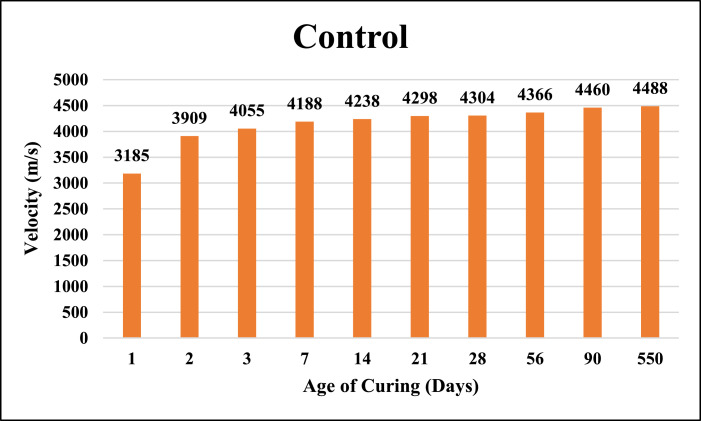
Average UPV of the Control mixture.

**Fig. 6:**
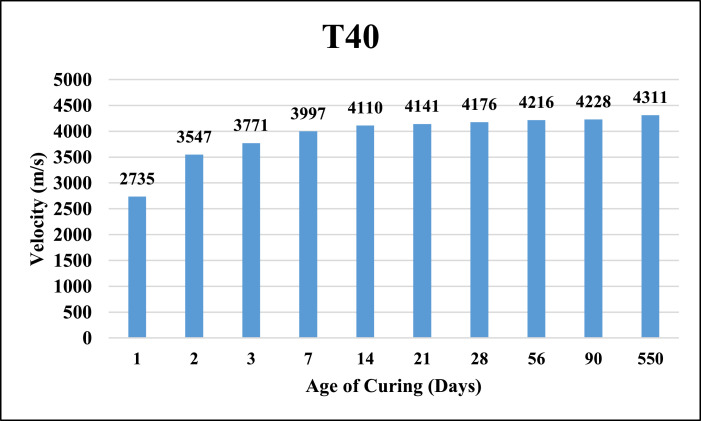
Average UPV of T40 mixture.

**Fig. 7:**
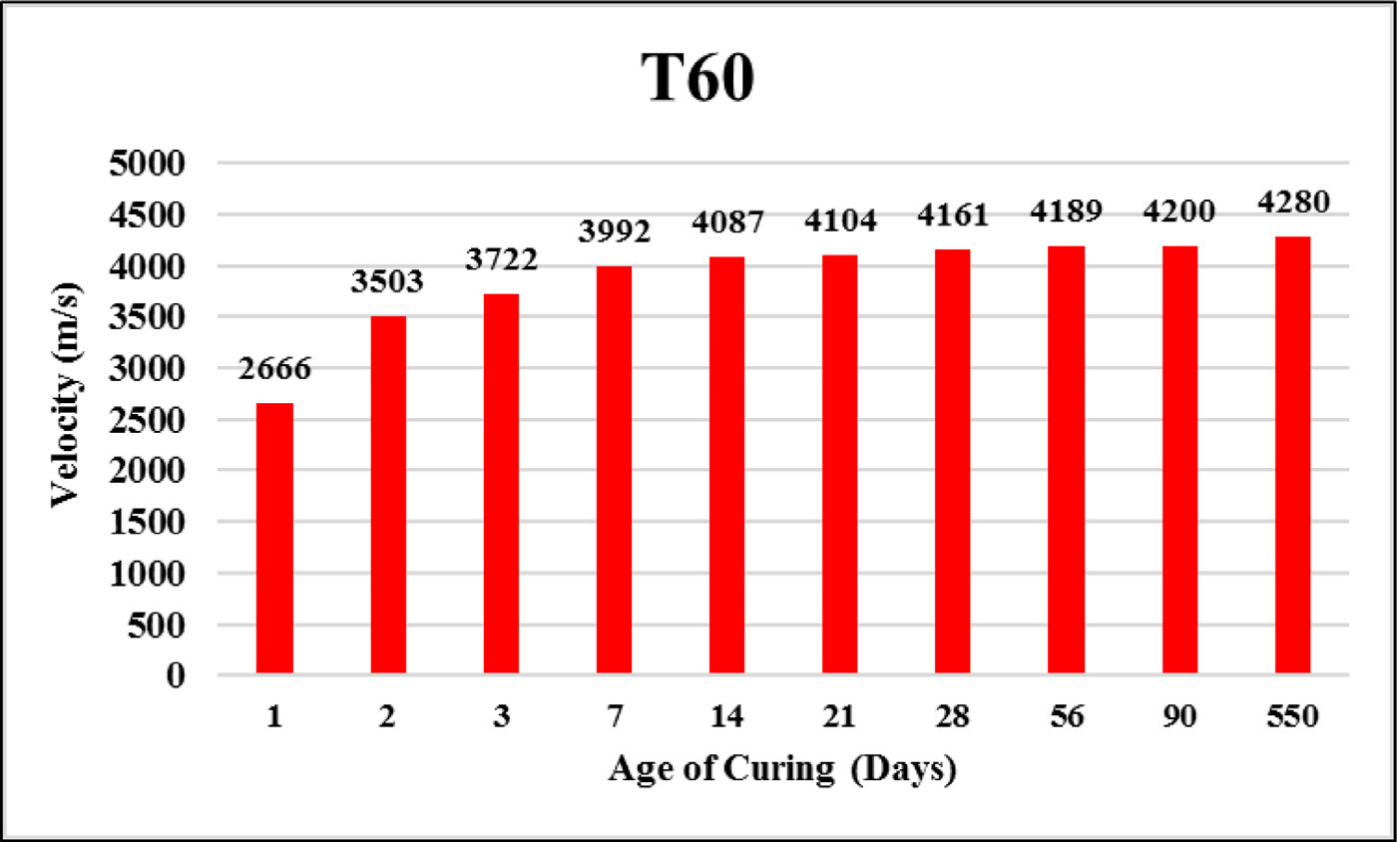
Average UPV of T60 mixture.

**Fig. 8:**
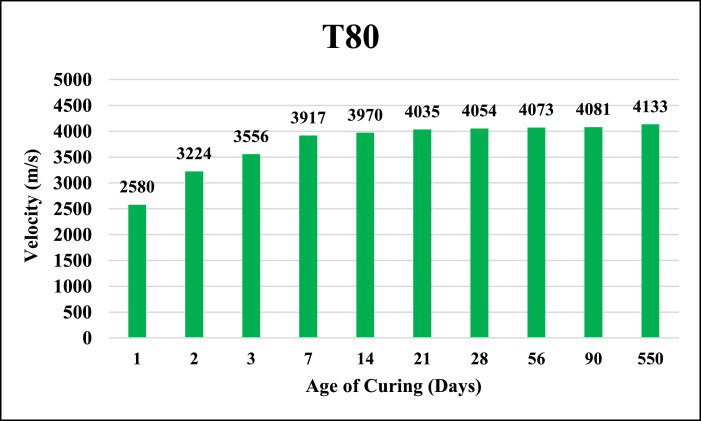
Average UPV of T80 mixture.

The dataset provided here represented the information for examining the compressive strength and ultrasonic pulse velocity (UPV) properties of the hardened mortar containing different combinations of Ground Granulated Blast Furnace Slag (GGBFS) and cement Kiln Dust (CKD) (in a high volume fraction) as alternatives of cement. Four mixtures were implemented, Control (reference mix without replacement) and three other mixtures included replacing the cement (by weight) with GGBFS and CKD combinations which designated as follows: T40 (26.7% GGBFS +13.3% CKD), T60 (40% GGBFS + 20% CKD), T80 (53.3% GGBFS + 26.7% CKD). The details of these mixtures can be found in [1]. The compressive strength and UPV tests were examined at 1, 2, 3, 7, 14, 21, 28, 56, 90 and 550 days of curing. The test results for Control, T40, T60 and T80 mixtures respectively are shown in Tables 1, 2, 3 and 4 (as well as in Figs. 1, 2, Fig. 3, Fig. 4) for compressive strength and in Tables 5, 6, 7 and 8 (as well as in Figs. 5, 6, Fig. 7, Fig. 8) for UPV. More detailed information about the compressive strength and UPV data can be found in the supplementary Excel datasets and in Ref. [1].

**Table 1:** Results of the compressive strength (MPa) for the Control mixture.

	**1 day**	**2 days**	**3 days**	**7 days**	**14 days**	**21 days**	**28 days**	**56 days**	**90 days**	**550 days**
**Sample 1**	8.75	17.1	23.34	30.67	35.84	35.81	40.14	38.82	40.7	44.24
**Sample 2**	8.41	16.9	23.25	34.52	35.72	37.41	37.47	39.63	40.8	44.31
**Sample 3**	7.97	17.2	22.03	34.03	36.11	36.92	34.55	39	40.9	44.16
**Sample 4**	8.47	17.3	24.97	33.2	35.91	37.42	37.42	39.22	40.4	44.26
**Average**	**8.40**	**17.13**	**23.40**	**33.11**	**35.90**	**36.89**	**37.40**	**39.17**	**40.70**	**44.24**

**Table 2:** Results of the compressive strength (MPa) for T40 mixture.

	**1 day**	**2 days**	**3 days**	**7 days**	**14 days**	**21 days**	**28 days**	**56 days**	**90 days**	**550 days**
**Sample 1**	10.51	10.22	10.91	23.21	29.33	32.27	39.11	39.23	39.12	45.21
**Sample 2**	9.37	11.40	11.65	22.89	28.64	33.10	37.07	38.24	40.14	44.12
**Sample 3**	9.61	9.96	10.39	22.73	27.60	31.64	39.06	38.17	41.30	43.60
**Sample 4**	9.47	9.36	10.47	23.04	26.98	31.44	37.30	38.30	40.13	43.78
**Average**	**9.74**	**10.24**	**10.86**	**22.97**	**28.14**	**32.11**	**38.14**	**38.49**	**40.17**	**44.18**

**Table 3:** Results of the compressive strength (MPa) for T60 mixture.

	**1 day**	**2 days**	**3 days**	**7 days**	**14 days**	**21 days**	**28 days**	**56 days**	**90 days**	**550 days**
**Sample 1**	4.67	6.84	9.58	23.18	23.88	29.88	36.94	36.8	40.62	43.41
**Sample 2**	3.95	7.81	8.93	22.64	24.87	28.96	35.97	37.62	39.75	43.52
**Sample 3**	4.74	7.4	9.74	22.34	24.76	29.55	37.77	37.47	39.62	41.34
**Sample 4**	4.94	7.29	10.11	22.22	25.12	30.02	36.75	38.72	40.33	41.42
**Average**	**4.58**	**7.34**	**9.59**	**22.60**	**24.66**	**29.60**	**36.86**	**37.65**	**40.08**	**42.42**

**Table 4:** Results of the compressive strength (MPa) for T80 mixture.

	**1 day**	**2 days**	**3 days**	**7 days**	**14 days**	**21 days**	**28 days**	**56 days**	**90 days**	**550 days**
**Sample 1**	3.21	6.80	8.94	22.11	23.40	25.11	26.14	33.17	33.29	34.42
**Sample 2**	3.22	6.41	9.40	21.80	22.14	24.66	27.11	33.28	33.17	33.12
**Sample 3**	3.08	7.40	8.94	21.62	21.88	26.10	25.18	32.98	33.30	33.40
**Sample 4**	3.08	6.70	9.40	21.44	22.11	24.12	26.12	33.19	33.33	33.16
**Average**	**3.15**	**6.83**	**9.17**	**21.74**	**22.38**	**25.00**	**26.14**	**33.16**	**33.27**	**33.53**

**Table 5:** Results of the UPV (m/s) for the Control mixture.

	**1 day**	**2 days**	**3 days**	**7 days**	**14 days**	**21 days**	**28 days**	**56 days**	**90 days**	**550 days**
**Sample 1**	3175	3891	4032	4201	4202	4292	4310	4356	4453	4478
**Sample 2**	3181	3922	4082	4190	4238	4298	4304	4374	4478	4490
**Sample 3**	3195	3912	4055	4182	4237	4310	4292	4358	4452	4480
**Sample 4**	3187	3910	4051	4178	4274	4292	4310	4376	4456	4502
**Average**	**3185**	**3909**	**4055**	**4188**	**4238**	**4298**	**4304**	**4366**	**4460**	**4488**

**Table 6:** Results of the UPV (m/s) for T40 mixture.

	**1 day**	**2 days**	**3 days**	**7 days**	**14 days**	**21 days**	**28 days**	**56 days**	**90 days**	**550 days**
**Sample 1**	2718	3551	3757	3974	4104	4122	4167	4216	4229	4299
**Sample 2**	2735	3543	3775	3997	4110	4128	4174	4226	4232	4317
**Sample 3**	2738	3547	3771	4022	4115	4172	4172	4214	4224	4311
**Sample 4**	2750	3548	3781	3995	4110	4140	4190	4211	4229	4317
**Average**	**2735**	**3547**	**3771**	**3997**	**4110**	**4141**	**4176**	**4216**	**4228**	**4311**

**Table 7:** Results of the UPV (m/s) for T60 mixture.

	**1 day**	**2 days**	**3 days**	**7 days**	**14 days**	**21 days**	**28 days**	**56 days**	**90 days**	**550 days**
**Sample 1**	2641	3509	3721	3984	4087	4082	4149	4191	4197	4282
**Sample 2**	2688	3497	3721	4000	4082	4104	4162	4196	4199	4292
**Sample 3**	2666	3502	3733	3992	4098	4149	4149	4188	4201	4292
**Sample 4**	2668	3503	3714	3992	4082	4082	4184	4182	4202	4254
**Average**	**2666**	**3503**	**3722**	**3992**	**4087**	**4104**	**4161**	**4189**	**4200**	**4280**

**Table 8:** Results of the UPV (m/s) for T80 mixture.

	**1 day**	**2 days**	**3 days**	**7 days**	**14 days**	**21 days**	**28 days**	**56 days**	**90 days**	**550 days**
**Sample 1**	2579	3234	3540	3934	3945	4024	4051	4072	4084	4118
**Sample 2**	2593	3221	3564	3899	3969	4034	4053	4073	4078	4130
**Sample 3**	2581	3221	3560	3924	3991	4039	4028	4074	4084	4152
**Sample 4**	2566	3220	3561	3912	3974	4042	4082	4074	4079	4134
**Average**	**2580**	**3224**	**3556**	**3917**	**3970**	**4035**	**4054**	**4073**	**4081**	**4133**

## 2 Experimental Design, Materials, and Methods

**Fig. 9:**
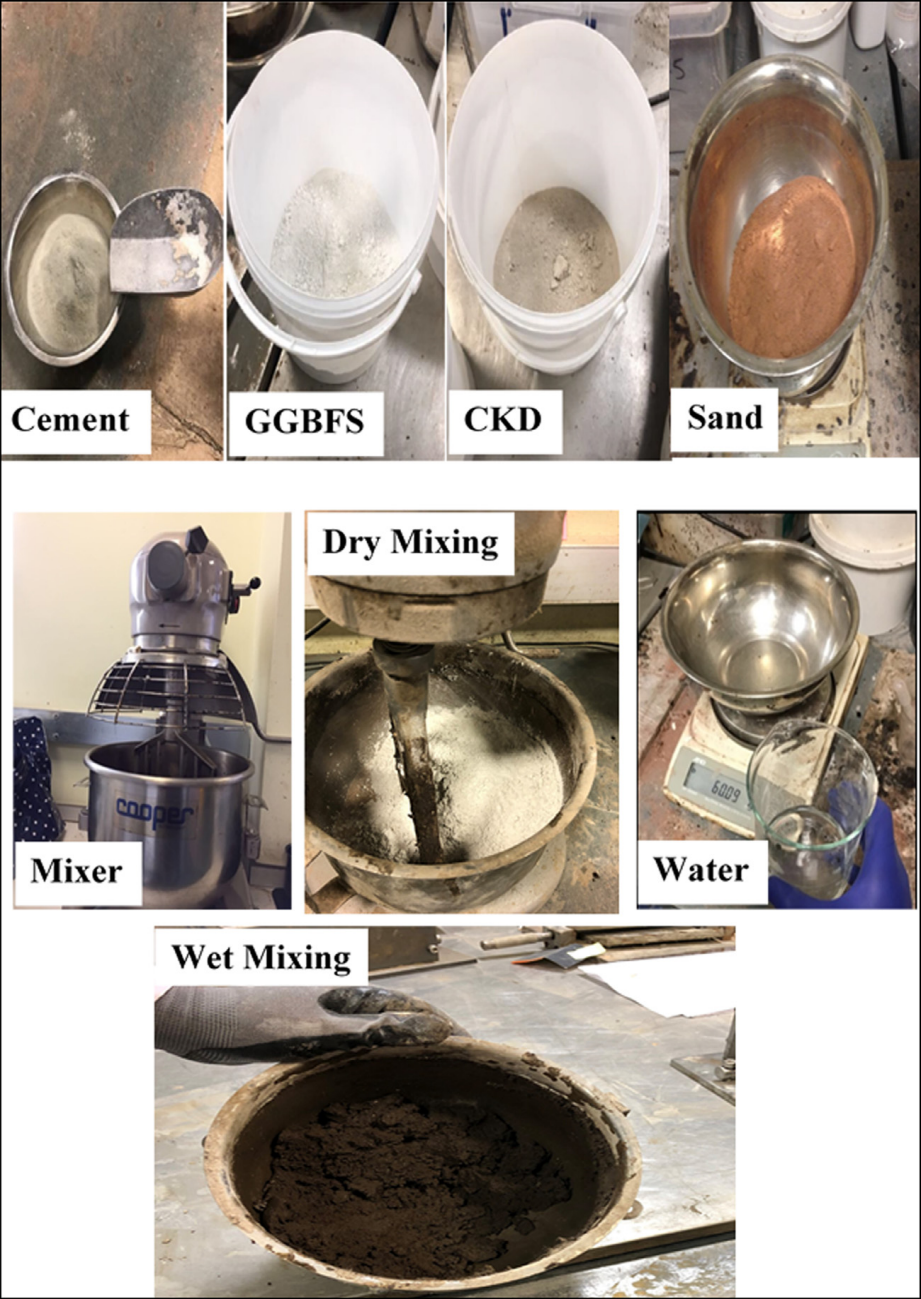
Raw materials and mixing of components for the preparation of samples.

**Fig. 10:**
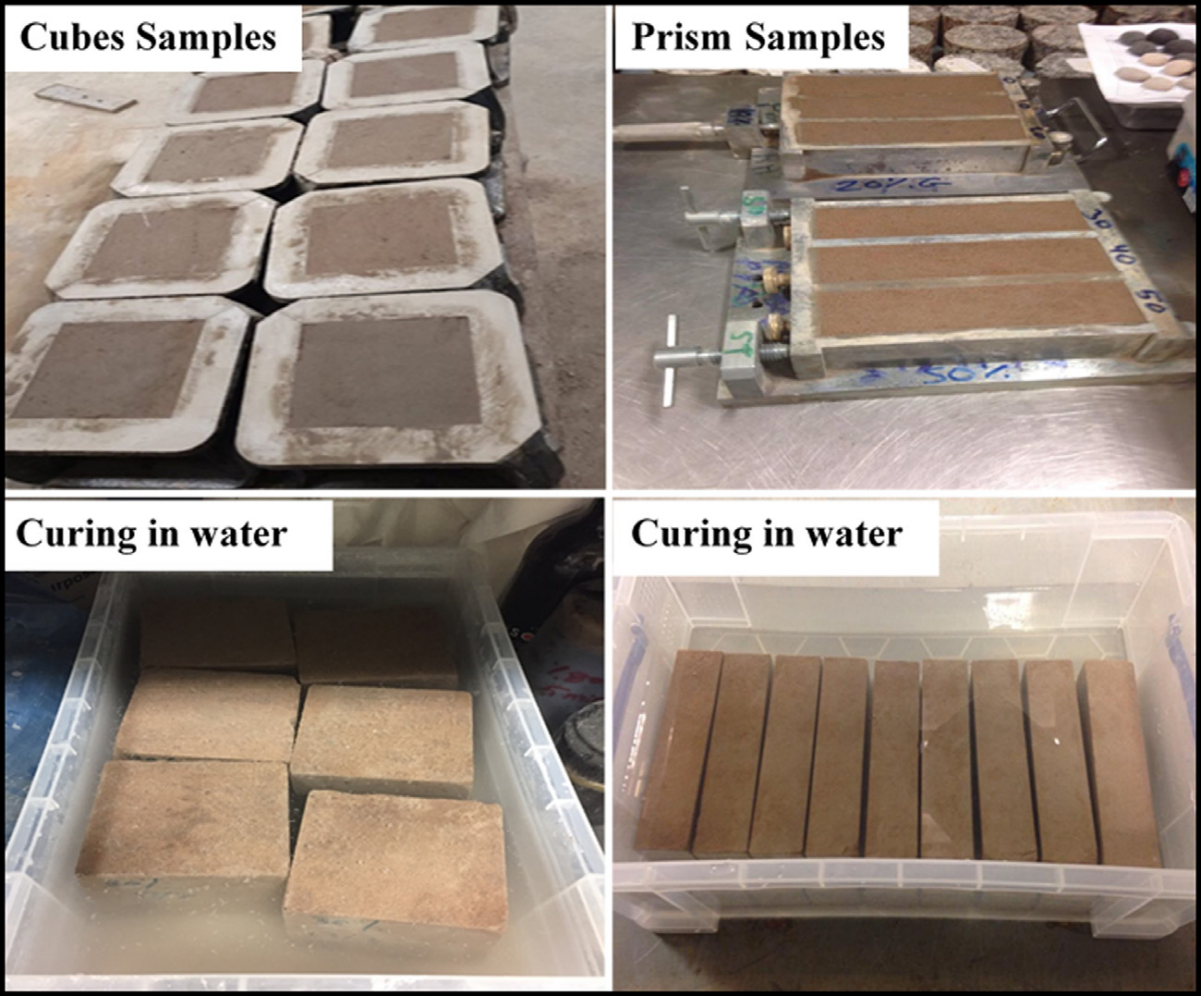
Cubes and prism samples in the moulds and curing in water after demoulding.

**Fig. 11:**
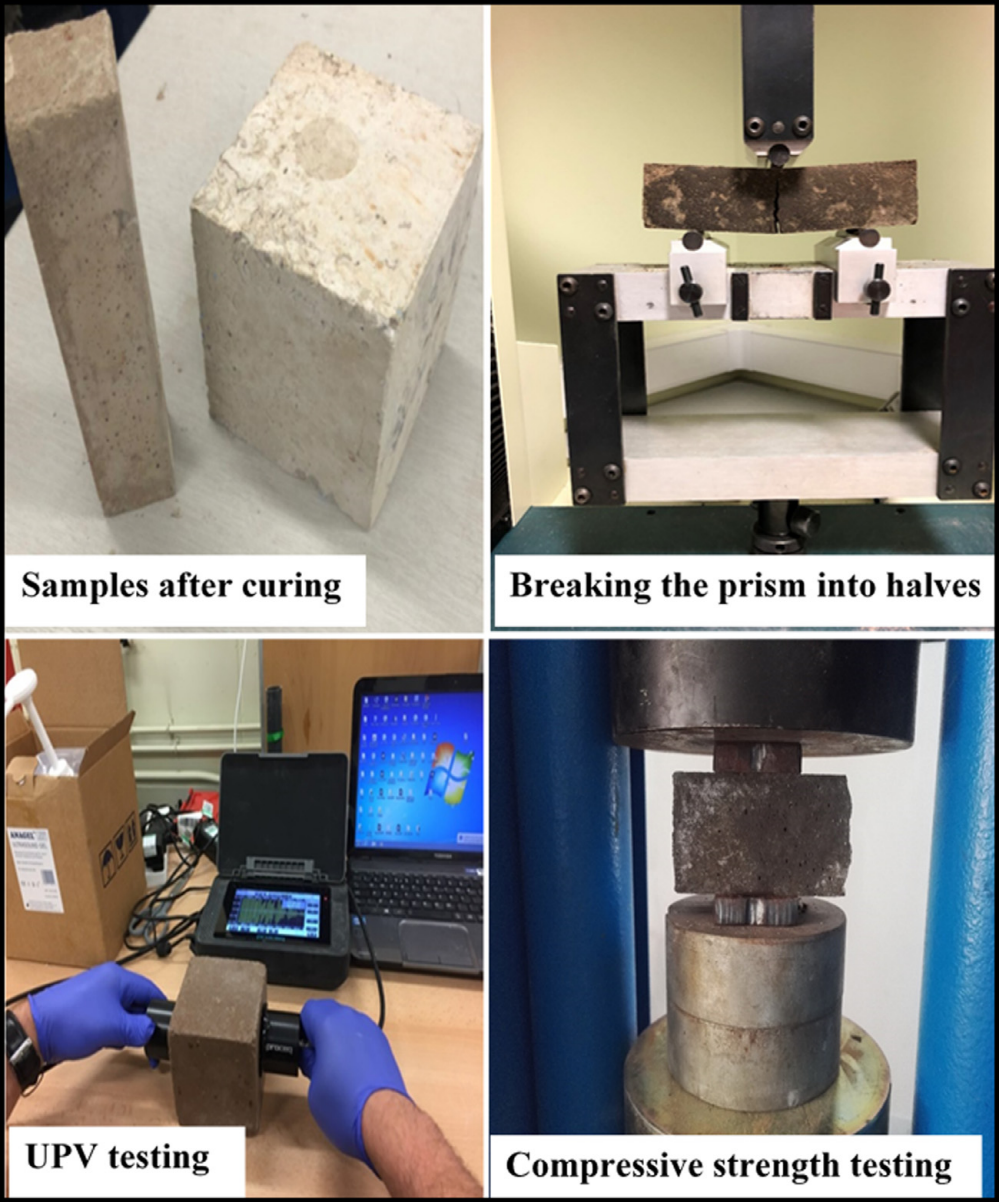
State of samples before testing and experimental setups of the UPV and compressive strength tests.

The main aim of using GGBFS and CKD as cement replacement materials is to reduce the environmental burden of cement manufacturing. For example, the cement industry consumes high energy as well as emits a high amount of CO_2_ into the atmosphere [2], [3], [4], [5], [6], [7]. The cement industry contributes about 7% of CO_2_ production worldwide [8], [9], [10], [11], [12], [13]. The laboratory work was conducted through the utilisation of different combinations of these materials in the production of mortar i.e no course aggregate was used in all mixtures. For all mixtures, the water to binder (W/B) ratio and sand to binder (S/B) ratio was fixed as 0.4 and 2.5, respectively. The GGBFS/CKD ratio in all the investigated mixtures was 2. The mortars were cast in 100 × 100 × 100 mm cubes for UPV measurements according to BS 1881-203 [14] while the prism moulds with the dimensions of 40 × 40 × 160 mm were used for compressive strength measurements according to BS EN 196-1 [15]. More data (images) about the method of mixing, preparation of samples, curing, state of samples before testing and experimental setups of the UPV and compressive strength tests are illustrated in Figs. 9 to 11.

## Declaration of Competing Interest

None.

## Appendix A Supplementary materials

Supplementary Data S1 Supplementary Raw Research Data. This is open data under the CC BY license http://creativecommons.org/licenses/by/4.0/ Supplementary Data S1
